# Self-Adaptive Image Thresholding within Nonextensive Entropy and the Variance of the Gray-Level Distribution

**DOI:** 10.3390/e24030319

**Published:** 2022-02-23

**Authors:** Qingyu Deng, Zeyi Shi, Congjie Ou

**Affiliations:** Department of Physics, College of Information Science and Engineering, Huaqiao University, Xiamen 361021, China; dqy595627951@163.com (Q.D.); shizeyi1@163.com (Z.S.)

**Keywords:** image thresholding, nonextensive entropy, Otsu-based algorithm, gray-level distribution, self-adaptive algorithm

## Abstract

In order to automatically recognize different kinds of objects from their backgrounds, a self-adaptive segmentation algorithm that can effectively extract the targets from various surroundings is of great importance. Image thresholding is widely adopted in this field because of its simplicity and high efficiency. The entropy-based and variance-based algorithms are two main kinds of image thresholding methods, and have been independently developed for different kinds of images over the years. In this paper, their advantages are combined and a new algorithm is proposed to deal with a more general scope of images, including the long-range correlations among the pixels that can be determined by a nonextensive parameter. In comparison with the other famous entropy-based and variance-based image thresholding algorithms, the new algorithm performs better in terms of correctness and robustness, as quantitatively demonstrated by four quality indices, ME, RAE, MHD, and PSNR. Furthermore, the whole process of the new algorithm has potential application in self-adaptive object recognition.

## 1. Introduction

One of the most important tasks in image segmentation is to precisely extract objects from their backgrounds. Image thresholding has previously been widely adopted because of its simplicity and efficiency [1,2,3]. For different types of images, a large number of thresholding algorithms exist based on the characteristics of images. More specifically, the gray-level distribution, i.e., the histogram of the gray-level image, plays an important role in the image thresholding algorithms. It is obvious that different types of images will show different histogram profiles, which contain information relating to both the objects and their backgrounds. Therefore, it is desirable to identify characteristic functions that can suggest proper thresholds to separate the objects and backgrounds.

The Otsu algorithm [4] is widely adopted to deal with images having a bimodal histogram distribution and can be easily extended to multi-level image segmentation [5,6,7,8,9]. The entropy-based algorithm [10,11,12,13,14] is another option for image segmentation since the gray-level histogram can be considered as a kind of probability distribution, and maximization of the corresponding entropies is a nature-inspired means of finding the optimal thresholds. In order to improve the robustness and anti-interference of the thresholding algorithms, two-dimensional histogram distributions [15,16,17] are frequently used to detect the edges and noise of the images, and thus achieve better segmentation results [18,19,20]. It is worth mentioning that, among these entropy-based algorithms, “Tsallis entropy-based thresholding” introduces the concept of nonextensivity into the image segmentation field [21,22]. The nonextensive entropy can be traced from the complex physical systems that have long-range interactions and/or long-duration memories [23,24]. There is a nonextensive parameter that measures the strength of the above mentioned non-local effects. Therefore, it is reasonable to adopt the nonextensive parameter to illustrate the global correlations among all pixels of an image. From the viewpoint of information theory, the nonextensive parameter of an image can be determined by the maximization of the redundancy of the gray-level distribution [25].

Since there are too many categories of images, a unique segmentation algorithm to deal with all of them effectively does not exist. Nevertheless, a stable algorithm that can correctly segment a wider variety of images is one of the important goals in computer vision research [26,27,28,29,30,31,32,33]. The Otsu and Otsu-based algorithms tend to separate the foreground and background equivalently, so they are not suitable for the extraction of tiny objects. Conversely, the entropy-based algorithms are too sensitive to the perturbation in images, and this instability restricts these algorithms from being applied in a more general scope. In this study, based on the explicit mathematical interpretation and numerical evaluation, it was found that the Otsu algorithm and the nonextensive entropy-based algorithm can be properly combined. An effective objective function is thus proposed to overcome both of the above-mentioned deficiencies. Moreover, the effective nonextensive parameter in the proposed algorithm is automatically determined by the information redundancy of an image [25]. Therefore, the proposes approach is a self-adaptive algorithm that can hopefully be applied to a more general scope of scenes.

The remainder of this paper is organized as follow: in Section 2, the general properties of the Otsu algorithm are illustrated, and the entropy-based algorithms are briefly introduced; in Section 3, based on mathematical calculation and numerical evaluation, an effective objective function is proposed for self-adaptive image thresholding; the detailed results and analysis are illustrated in Section 4; and the conclusions are presented in Section 5.

## 2. Image Thresholding Algorithms

Assuming that the size of an image is M×N and the range of its gray-level is considered as i=0,1,…,L−1, the probability of the i-th gray-level can be defined as:

(1) $$p_{i}=\frac{h_{i}}{M\times N},p_{i}\geq 0,\sum_{i=1}^{L}p_{i}=1$$

where M×N is the total number of pixels in the image, $h_{i}$ represents the number of pixels for which the gray-level value is equal to i. Therefore, the normalization of the probability distribution is explicitly expressed as Equation (1).

### 2.1. Otsu Algorithm

Now suppose that the threshold of an image is t. The corresponding gray-level histogram can be divided into two classes, $C_{a}=(0,1,\ldots ,t)$ and $C_{b}=(t+1,\ldots ,L-1)$, and the cumulative probability of the above two classes can be written as:

(2) $$P_{a}=\sum_{i=0}^{t}p_{i},P_{b}=\sum_{i=t+1}^{L-1}p_{i}$$

The mean values of the gray-level of $C_{a}$ and $C_{b}$ are given by:

(3) $$\left\{ \begin{array}{l} \omega_{a}=\frac{1}{P_{a}}\sum_{i=0}^{t}ip_{i}\\ \omega_{b}=\frac{1}{P_{b}}\sum_{i=t+1}^{L-1}ip_{i} \end{array}\right.$$

Using the same idea, the mean gray-level value of the image is:

(4) $$\omega_{G}=\sum_{i=0}^{L-1}ip_{i}$$

Therefore, the variances of $C_{a}$, $C_{b}$, and the total histogram can be respectively written as:

(5) $$\left\{ \begin{array}{l} \sigma_{a}^{2}=\sum_{i=0}^{t}(i-\omega_{a})\frac{p_{i}}{P_{a}},\sigma_{b}^{2}=\sum_{i=t+1}^{L-1}(i-\omega_{b})\frac{p_{i}}{P_{b}}\\ \sigma_{G}^{2}=\sum_{i=0}^{L-1}(i-\omega_{G})p_{i} \end{array}\right.$$

Based on Equation (5), the within-class variance and between-class variance are defined as [4]:

(6) $$\left\{ \begin{array}{l} \sigma_{W}^{2}=\omega_{a}\sigma_{a}^{2}+\omega_{b}\sigma_{b}^{2}\\ \sigma_{B}^{2}=P_{a}{\left(\omega_{a}-\omega_{G}\right)}^{2}+P_{b}{\left(\omega_{b}-\omega_{G}\right)}^{2} \end{array}\right.$$

and the following relation holds:

(7) $$\sigma_{B}^{2}+\sigma_{W}^{2}=\sigma_{G}^{2}$$

It can be easily seen that for the arbitrary threshold value t, the following relations always hold:

(8) $$\left\{ \begin{array}{l} P_{a}\omega_{a}+P_{b}\omega_{b}=\omega_{G}\\ P_{a}+P_{b}=1 \end{array}\right.$$

The key point of the Otsu algorithm is to maximize the between-class variance by selecting a proper threshold value $t^{*}$, i.e.,

(9) $$t^{*}=Arg\max\{\sigma_{B}^{2}(t)\}$$

In fact, using Equation (8), the between-class variance can be rewritten as:

(10) $$\sigma_{B}^{2}=P_{a}P_{b}{\left(\omega_{b}-\omega_{a}\right)}^{2}$$

From Equation (10), it is shown that the between-class variance is dominated by two factors, ${\left(\omega_{b}-\omega_{a}\right)}^{2}$ and $P_{a}P_{b}$. Maximizing the factor ${\left(\omega_{b}-\omega_{a}\right)}^{2}$ means that the gray-level difference between $C_{a}$ and $C_{b}$ is tuned to the maximum by a proper threshold $t_{1}$, which coincides with the principle of image segmentation. However, maximizing the factor $P_{a}P_{b}$ requires finding another threshold $t_{2}$ to satisfy $P_{a}=P_{b}=1/2$, which means that the number of pixels in the foreground is equal to that in the background. In general, for a given image, $t_{1}=t_{2}$. However, the optimal threshold $t^{*}$ represents the trade-off between $t_{1}$ and $t_{2}$. Therefore, the Otsu algorithm always has a tendency to equally separate the pixels of an image, demonstrating the deficiency when extracting tiny objects from the background.

### 2.2. Otsu–Kapur Algorithm

The Otsu algorithm is a classical global thresholding technique based on the clustering theorem. The idea of the entropy-based algorithm is quite different from Otsu’s, although both of them start from the image’s histogram. Shannon entropy is widely adopted in entropy-based image thresholding. It was first proposed by Pun [34] and improved by Kapur in 1985 [35]. By using the a priori entropy of the foreground and background, an objective function is obtained to indicate the optimal threshold under the Maximum Entropy Principle.

Based on the gray-level histogram distribution of an image, Shannon entropy is given by:

(11) $$S_{k}=-\sum_{i=0}^{L-1}p_{i}\ln p_{i}$$

Assuming that the histogram is separated into two parts (a and b) by threshold t,. the corresponding entropies are:

(12) $$\left\{ \begin{array}{l} S(a)=-\sum_{i=0}^{t}\frac{p_{i}}{P_{t}}\ln\frac{p_{i}}{P_{t}}=\ln P_{t}+\frac{S_{t}}{P_{t}}\\ S(b)=-\sum_{i=t+1}^{L-1}\frac{p_{i}}{1-P_{t}}\ln\frac{p_{i}}{1-P_{t}}=\ln(1-P_{t})+\frac{S_{k}-S_{t}}{1-P_{t}}\\ P_{t}=\sum_{i=0}^{t}p_{i},\;S_{t}=-\sum_{i=0}^{t}p_{i}\ln\frac{p_{i}}{P_{t}} \end{array}\right.$$

The objective function φ(t) is given by the sum of S(a) and S(b):

(13) φ(t)=S(a)+S(b)

and the optimal threshold of Kapur algorithm is determined by:

(14) $$t^{*}=Arg\max\{\varphi (t)\}$$

In practice, the Kapur algorithm has better performance than the Otsu algorithm in extracting tiny targets from their background. However, this algorithm is quite sensitive to the perturbation of pixels. For instance, the value of Equation (13) varies drastically with the threshold t, which means that the optimal threshold can be easily disturbed by the variation in gray-level distribution and lead to incorrect segmentation. This instability also restricts the application of the Kapur algorithm to a more general scope. Taking the characteristics of the Otsu algorithm into account, it is possible to increase the stability by combining the Kapur and Otsu algorithms, without losing the accuracy of extracting tiny objects.

For a given image, the total gray-level variance $\sigma_{G}^{2}$ is fixed. From Equation (7), we can see that maximizing the between-class variance p(x) is equivalent to minimizing the within-class variance $\sigma_{W}^{2}$. Therefore, Equations (5) and (14) yield the objective function:

(15) $$N_{e}(t)=\ln\sigma_{W}{\left(t\right)}^{2}-\varphi (t)$$

The optimal threshold is obtained by the following algorithm:

(16) $$t^{*}=Arg\min\{N_{e}(t)\}$$

### 2.3. Two-Dimensional Entropic Algorithm

The above-mentioned thresholding algorithms are based on the one-dimensional(1D) gray-level histogram. In order to improve the accuracy and robustness, Ahmed [36] considered not only the pixel’s gray-level value, but also the spatial correlation of the pixels in an image. Therefore, the mean gray-level value of neighboring pixels is relevant and the one-dimensional (1D) histogram distribution is extended to the two-dimensional (2D) distribution. If a pixel’s gray-level is equal to i and the average gray-level of its neighborhood is j, the number of this kind of pixel among the image is $f_{ij}$.

The 2D probability distribution can be written as:

(17) $$p_{ij}=\frac{f_{ij}}{M\times N}$$

The total entropy of the 2D histogram is defined as:

(18) $$H(L)=-\sum_{i=0}^{L-1}\sum_{j=0}^{L-1}p_{ij}\ln p_{ij}$$

**Figure 1:**
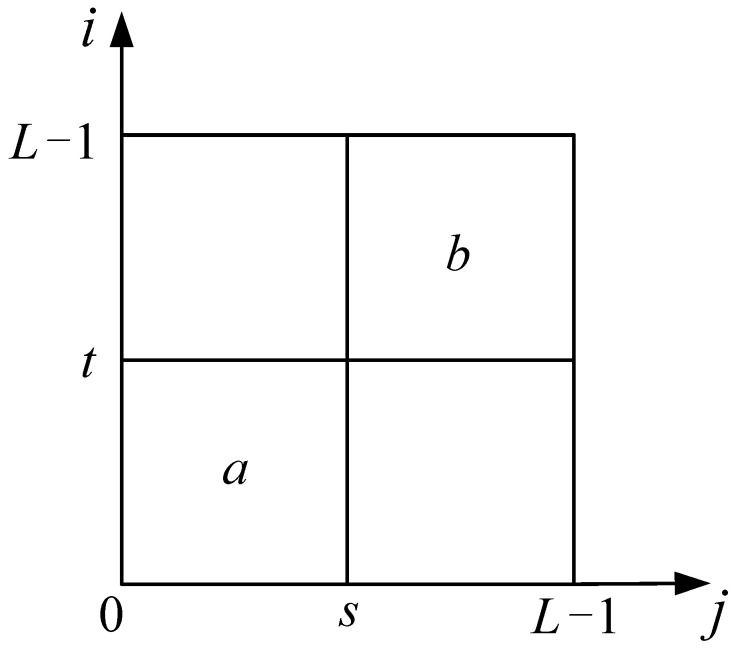
The distribution of the two-dimensional histogram with the threshold (*s*, *t*).

If the two thresholds are located at s and t, the 2D gray-level histogram is divided into four regions, as shown in Figure 1.

Assume that the pixels are mainly distributed at two regions, a and b in Figure 1. The cumulative probabilities a of and b are:

(19) $$\left\{ \begin{array}{l} P_{A}(s,t)=\sum_{i=0}^{s}\sum_{j=0}^{t}p_{ij}\\ P_{B}(s,t)=\sum_{i=s+1}^{L-1}\sum_{j=t+1}^{L-1}p_{ij} \end{array}\right.$$

The corresponding entropies can be written as:

(20) $$\left\{ \begin{array}{l} H_{A}(s,t)=-\sum_{i=0}^{s}\sum_{j=0}^{t}\frac{p_{ij}}{P_{A}(s,t)}\ln\frac{p_{ij}}{P_{A}(s,t)}\\ H_{B}(s,t)=-\sum_{i=s+1}^{L-1}\sum_{j=t+1}^{L-1}\frac{p_{ij}}{P_{B}(s,t)}\ln\frac{p_{ij}}{P_{B}(s,t)} \end{array}\right.$$

Based on the additivity of Shannon entropy, the total entropy is defined as:

(21) $$\Psi (s,t)=H_{A}(s,t)+H_{B}(s,t)$$

which is dependent on threshold (s,t). By the same idea of the 1D entropy-based algorithm, maximizing the objective function, i.e., Equation (21), can yield the optimal thresholds:

(22) $$(s^{*},t^{*})=Arg\left\{\underset{0<s<L-1}{\max}\left\{\underset{0<t<L-1}{\max}\Psi \left(s,t\right)\right\}\right\}$$

In practice, the above 2D entropic algorithm is effective for images with uneven illumination, noise, missing edges, poor contrast, and other interference from the environment [37]. It is reasonable to consider more correlations between the pixel and its neighborhood, and the histogram distribution can be extended to higher dimensions. However, the increase in the number of dimensions will lead to an exponential increment in computation.

### 2.4. Tsallis Entropy Algorithm

As mentioned above, Shannon entropy is additive and shows the property of extensivity in image processing. The concept of entropy was first proposed in thermodynamics to describe the physical systems that have a huge number of microstates. Furthermore, the extensivity of entropy is based on the assumption that the microstates among the system are independent of each other. However, for some systems with long-range interactions, long-time memory and fractal-type structures, the extensivity may not hold anymore. Tsallis introduces a kind of non-extensive entropy [23] to describe such systems, expressed as:

(23) $$S_{T}\equiv \frac{1-\sum_{i=1}^{L}p_{i}^{q}}{q-1}$$

where q is a real number that describes the nonextensivity of the system. In the q→1 limit, Tsallis entropy is reduced to Shannon entropy and the extensivity of the system is recovered. The nonextensive generalization of entropy also shed lights on the information theory. In image segmentation, Tsallis entropy shows potential superiority and flexibility in a more general scope of image class [21].

In the Tsallis entropy algorithm, the cumulative probability of foreground a and background b are:

(24) $$P_{a}=\sum_{i=1}^{t}p_{i},P_{b}=\sum_{i=t+1}^{L}p_{i}$$

According to Equation (23), the entropy of each part can be defined as [21]:

(25) $$\left\{ \begin{array}{l} S_{T}^{a}(t)=\frac{1-\sum_{i=1}^{t}{\left(\frac{p_{i}}{P_{a}}\right)}^{q}}{q-1}\\ S_{T}^{b}(t)=\frac{1-\sum_{i=t+1}^{L}{\left(\frac{p_{i}}{P_{b}}\right)}^{q}}{q-1} \end{array}\right.$$

Suppose that a and b are subsystems of the full image, due to the nonextensivity; the total entropy of the image is expressed as:

(26) $$S_{q}^{a+b}(t)=S_{q}^{a}(t)+S_{q}^{b}(t)+(1-q)S_{q}^{a}(t)S_{q}^{b}(t)$$

where the third term on the right-hand side of Equation (26) shows the pseudo-additivity of Tsallis entropy. Maximizing $S_{q}^{a+b}$ yields the optimal threshold $t^{*}$, which is given by:

(27) $$t^{*}=\arg\max\left\{S_{q}^{a+b}(t)\right\}$$

Obviously, the optimal result of Equation (27) depends on the nonextensive parameter q, which describes the strength of internal correlation of the image. In other words, for an arbitrary two pixels in the image, their gray-level values may have long-range correlations. More specifically, for an image containing several objects, the pixels of objects will exhibit similar gray-level values, even though they are not adjacent to each other. It is possible to measure this kind of long-range correlation by nonextensive entropy [18,21], and this idea inspired a new algorithm that is discussed below. Since the parameter q is an additional index that can tune the optimal threshold, it is of great importance to determine the exact value of q for a given image. Recently, Abdiel and coauthors introduce a methodology to evaluate the nonextensive parameter q of an image [25]. Based on the information theory, the generalized redundancy of an image that presents nonextensive properties can be expressed as [25]:

(28) $$R(q)=1-\frac{S_{T}}{S_{T\max}}$$

where $S_{T\max}=\left(1-L^{1-q}\right)/\left(q-1\right)$ is the possible maximum *q*-entropy of the image that can be achieved at $p_{i}=1/L(0\leq i\leq L-1)$, i.e., equipartition of the gray-level probability. Maximizing Equation (28) by a proper value of q means that the gray-level histogram of the given image is renormalized to deviate from the equal probability case (containing zero information) as far as possible. Therefore, the information contained in the image histogram can be strengthened by a particular q, which is highly image category dependent.

## 3. New Algorithm

As mentioned above, the nonextensive entropy algorithm is suitable for describing the long-range correlations within an image. However, like other entropy-based algorithms, it is still very sensitive to the perturbation of signals, so the scope of its application is limited. By comparison, the Otsu algorithm is stable but not accurate for small target extraction. Therefore, it is possible to combine the advantages of the two and develop a new algorithm with a more general scope of application. It is worth mentioning that the nonextensive parameter q in Tsallis entropy is now determined by information redundancy and cannot be tuned arbitrarily.

Based on Equations (5), (7) and (26), a new objective function can be written as:

(29) $$\mu (t)=S_{q}^{a+b}-{\left(\sigma_{W}^{2}\right)}^{1-q}$$

In order to retain the concavity of Tsallis entropy, q>0 should be satisfied [23]. Alternatively, q<1 is called superextensivity, which will increase the total entropy of the system in comparison with the extensive case (q=1) [38]. In practice, almost all categories of images exhibit the property of superextensivity [25]. Therefore, the proper range of the nonextensive parameter can be 0<q<1. From Equations (9) and (27), we can see that both of the two algorithms are aimed to maximize the objective functions. Taking Equation (7) into account, it can be easily seen that the aim of Equation (29) is to maximize the objective function, i.e.,

(30) $$t^{*}=Arg\max\{\mu (t)\}$$

**Figure 2:**
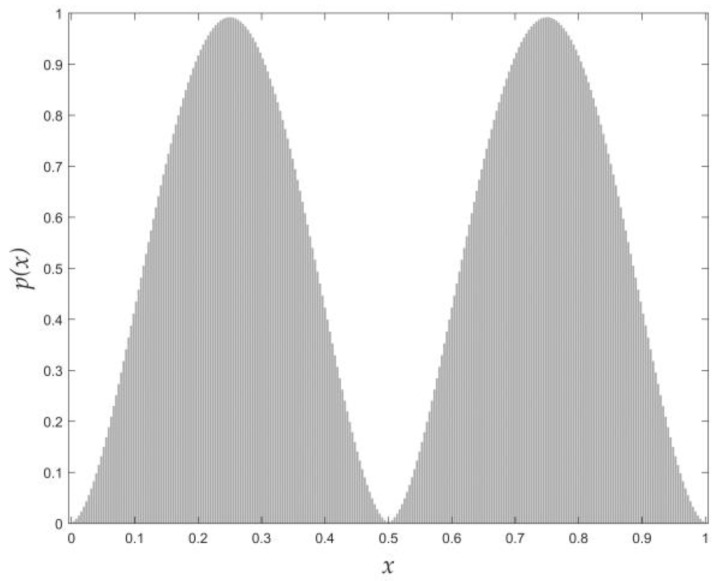
Normalized histogram distribution.

**Figure 3:**
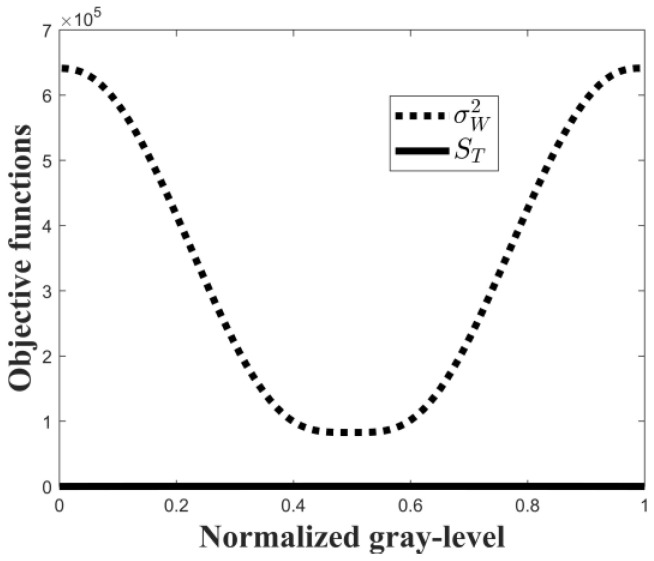
Objective functions of the Otsu and Tsallis algorithms.

The optimal threshold is obtained from Equation (30) with the above-mentioned range of q. For a synthetic image having a bimodal histogram distribution, as shown in Figure 2, the profile of each peak is the normalized *q*-Gaussian distribution function [39]. From Equations (9) and (27), we can see that both the Otsu algorithm and the Tsallis entropy algorithm indicate the valley gray-level between the two peaks as the optimal threshold, which exactly coincides with the result of Equation (30). For other natural pictures that have an arbitrary histogram distribution, there is no evidence that the result of Equation (9) coincides with that of Equation (27), whereas Equation (29) shows a trade-off between them and Equation (30) may yield a proper suggestion. For the histogram of Figure 2, it should be noted that the magnitude difference between $S_{q}^{a+b}$ and $\sigma_{W}^{2}$ is very large. As shown in Figure 3, both of them are functions of threshold t. However, the values of the Tsallis entropy algorithm are totally suppressed by those of the Otsu algorithm for any possible threshold t. Therefore, it is unsuitable to combine $S_{q}^{a+b}$ and $\sigma_{W}^{2}$ directly.

In order to avoid the impact of the magnitude difference, the *q*-exponential function [40] can be adopted to revise the magnitude of $\sigma_{W}^{2}$. By definition, Tsallis entropy with a continuous probability distribution function can be expressed as:

(31) $$S_{T}=\frac{1-\int_{0}^{1}p{\left(x\right)}^{q}dx}{q-1}$$

where p(x) represents the probability density of the normalized gray-level value x. For a system presenting nonextensive *q*-entropy, the corresponding probability distribution can be written as the *q*-Gaussian function [39]:

(32) $$p(x)=\frac{1}{Z_{q}}{\left[1-(1-q)\cdot \frac{x^{2}}{\sigma^{2}}\right]}^{\frac{1}{1-q}}$$

where $\sigma^{2}$ is the variance of x and $Z_{q}$ is the partition function to keep the probability normalization condition, i.e.,

(33) $$\begin{array}{ll} Z_{q} & =\int_{0}^{1}{\left[1-(1-q)\cdot {\left(\frac{x}{\sigma }\right)}^{2}\right]}^{\frac{1}{1-q}}dx\\ & =\frac{\sigma \sqrt{\pi }}{2\sqrt{1-q}}\cdot \frac{\Gamma \left(1+\frac{1}{1-q}\right)}{\Gamma \left(\frac{3}{2}+\frac{1}{1-q}\right)} \end{array}$$

where Γ(k) is the Gamma function and will reduce to factorial (k−1)! if k is an integer. Substituting p(x) into Equation (31) yields:

(34) $$\begin{array}{ll} S_{T} & =\frac{1-{\int_{0}^{1}\frac{1}{Z_{q}^{q}}\left[1-(1-q){\left(\frac{x}{\sigma }\right)}^{2}\right]}^{\frac{q}{1-q}}dx}{q-1}\\ & =\frac{1-\xi \cdot {\left(\sigma^{2}\right)}^{\frac{1-q}{2}}}{q-1} \end{array}$$

where:

(35) $$\xi ={\left[\frac{\pi }{4(1-q)}\right]}^{\frac{1-q}{2}}\cdot {\left[\frac{\Gamma \left(\frac{3}{2}+\frac{1}{1-q}\right)}{\Gamma \left(1+\frac{1}{1-q}\right)}\right]}^{q}\cdot \frac{\Gamma \left(\frac{1}{1-q}\right)}{\Gamma \left(\frac{3-q}{2(1-q)}\right)}$$

is the integration constant for a given value of *q*. If $p_{a}$ and $p_{b}$ are two identical *q*-Gaussian distribution functions, according to the nonextensivity of Tsallis entropy, the total entropy can be written as:

(36) $$\begin{array}{ll} S_{T}(a+b) & =S_{T}(a)+S_{T}(b)+(1-q)S_{T}(a)S_{T}(b)\\ & =\frac{1-\xi_{a}{\left(\sigma_{a}^{2}\right)}^{\frac{1-q}{2}}}{q-1}+\frac{1-\xi_{b}{\left(\sigma_{b}^{2}\right)}^{\frac{1-q}{2}}}{q-1}+\\ & (1-q)\cdot \frac{1-\xi_{a}{\left(\sigma_{a}^{2}\right)}^{\frac{1-q}{2}}}{q-1}\cdot \frac{1-\xi_{b}{\left(\sigma_{b}^{2}\right)}^{\frac{1-q}{2}}}{q-1} \end{array}$$

Substituting $\sigma_{a}^{2}=\sigma_{b}^{2}=\sigma_{W}^{2}$ into Equation (36) yields:

(37) $$S_{T}(a+b)=\frac{\xi_{a}\xi_{b}{\left(\sigma_{W}^{2}\right)}^{1-q}-1}{1-q}$$

**Figure 4:**
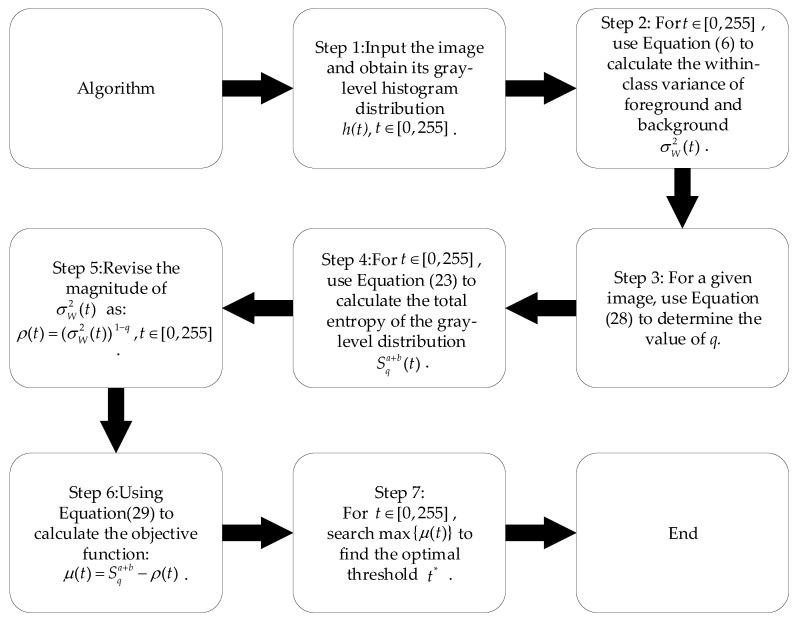
The procedure of the new algorithm.

Therefore, the magnitude of ${\left(\sigma_{W}^{2}\right)}^{1-q}$ is comparable with $S_{T}(a+b)$ at the proper range of *q*, and the rationality of Equation (29) is shown. The main steps of the present algorithm can be seen in Figure 4:

The above procedure can also be applied to the segmentation of RGB or other color images. The intense distribution of each color channel can be considered as a gray-level distribution. Therefore, the threshold value of each channel can be obtained directly. It should be mentioned that the intense distributions may differ for different color channels, so the above algorithm cannot yield a unified value, in general. By comparison, both the Otsu algorithm and entropy-based algorithm can be independently adopted for multi-level image thresholding. According to the idea of Equation (29), the advantages of these two kinds of typical thresholding algorithms can be combined by extending Equation (29) to the multi-level case.

## 4. Analysis of Experimental Results

In order to show the stability and feasibility of the proposed algorithm, we used four quality indices, namely, Misclassification Error (ME), Relative Foreground Area Error (RAE), Modified Hausdorff Distance (MHD), and Peak Signal-to-Noise Ratio (PSNR), to illustrate the performance of Equation (29) and make comparisons with the other algorithms mentioned in Section 2.

### 4.1. Misclassification Error (ME)

Misclassification error expresses the percentage of wrongly assigned image pixels that represent the object and background images. For the single threshold segmentation, ME can be simply expressed as [41]:

(38) $$ME=1-\frac{\left|C_{gt}\cap C_{t}\right|+\left|B_{gt}\cap B_{t}\right|}{\left|C_{gt}\right|+\left|B_{gt}\right|}$$

where $C_{gt}$ and $B_{gt}$ represent the foreground and background of the ground-truth image, $C_{t}$ and $B_{t}$ are the foreground and background pixels in the segmented image, and | . | is the cardinality of the set. The value of ME is between 0 and 1. The lower the value of ME, the better the segmentation result.

### 4.2. Relative Foreground Area Error (RAE)

RAE is a quality assessment parameter that calculates the area of difference between the segmented image and the ground-truth image, which is defined as [42]:

(39) $$RAE=\{\begin{array}{c} \frac{A_{s}-A_{t}}{A_{s}},\;ifA_{t}<A_{s}\\ \frac{A_{t}-A_{s}}{A_{t}},\;ifA_{s}<A_{t} \end{array}$$

where $A_{s}$ and $A_{t}$ are the area of the ground-truth image and the segmented image, respectively. Obviously, for an ideal segmentation in which $A_{t}$ coincides with $A_{s}$, RAE is zero.

### 4.3. Modified Hausdorff Distance (MHD)

Hausdorff distance is used to determine the degree of similarity between two objects that are overlapped with each other. In order to maintain the symmetry form, the Modified Hausdorff Distance (MHD) is more frequently used and is defined as [43]:

(40) $$MHD(R_{gt},R_{t})=\max(d_{MHD}(R_{gt},R_{t}),d_{MHD}(R_{t},R_{gt}))$$

(41) $$d_{MHD}(R_{gt},R_{t})=\frac{1}{R_{gt}}\sum_{r_{gt}\in R_{gt}}\underset{r_{t}\in R_{t}}{\min}\left\|r_{gt}-r_{t}\right\|$$

where $r_{gt}$ and $r_{t}$ represent objects belonging to the ground-truth image $R_{gt}$ and the segmented result $R_{t}$, respectively, and $\left\|r_{gt}-r_{t}\right\|$ is the Hausdorff distance. This parameter can objectively describe the distortion degree of the segmented image and the ground-truth image. If $R_{t}$ perfectly coincides with $R_{gt}$, then MHD is zero, by definition. Unlike ME and RAE, MHD is not normalized. For failed segmentation, the value of MHD will be much larger than 1.

### 4.4. Peak Signal-to-Noise Ratio (PSNR)

The Peak Signal-to-Noise Ratio is a measurement algorithm used in the image transmission. First, the concept of Mean Square Error (MSE) is required, which is a measure of the difference between two images. It is defined as [44]:

(42) $$MSE=\frac{1}{M\times N}\sum_{i=0}^{M-1}\sum_{j=0}^{N-1}{\left[R_{gt}(i,j)-R_{t}(i,j)\right]}^{2}$$

where $R_{gt}(i,j)$ and $R_{t}(i,j)$ are pixels of the ground-truth image and segmented image, respectively. It can be easily seen that MSE=0 if $R_{gt}(i,j)=R_{t}(i.j)$ for arbitrary coordinates (i,j). Therefore, lower MSE represents better quality of image segmentation. Accordingly, PSNR is defined in terms of MSE:

(43) $$PSNR=10\cdot \log_{10}\left(\frac{{\left(L-1\right)}^{2}}{MSE}\right)$$

Equation (43) shows that, for ideal segmentation (MSE→0), PSNR will tend to infinity.

### 4.5. Experimental Results

**Figure 5:**
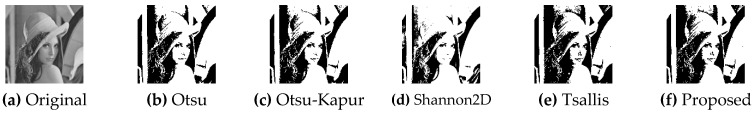
Lena.

**Figure 6:**
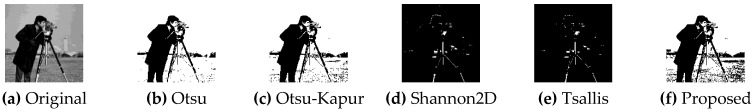
Cameraman.

**Figure 7:**
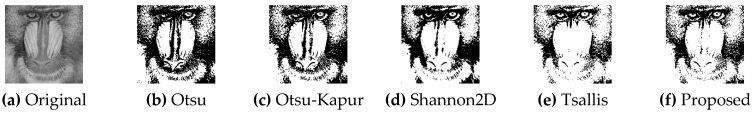
Baboon.

First, we applied the proposed algorithm to segment several well-known testing images. The results of the four other algorithms mentioned above are also listed, as shown in Figure 5, Figure 6 and Figure 7.

In Figure 5, compared to the results of the four other algorithms, i.e., Figure 5b–e, the result of the proposed algorithm has more details and edge contours. In Figure 6, we can see that both the 2D histogram algorithm and the Tsallis entropic algorithm failed to extract the objects from the image. However, the result of the proposed algorithm, i.e., Figure 6f is quite acceptable. We can see more detail information in it, in comparison with Figure 6b,c. In Figure 7e, it is shown that the Tsallis entropic algorithm over segments the original image and the detail of the baboon’s face is lost. However, the Otsu, Otsu–Kapur and Shannon 2D thresholds are also not appropriate. As shown in Figure 7b–d, the baboon’s eyes are blurred by too many black pixels. In contrast, the proposed algorithm has a moderate result, as shown in Figure 7f.

In order to show the advantages of the proposed algorithm more convincingly, we choose 50 test images from VOC-2012, BSD300, and Ref. [45] to compare the performance of these five algorithms. These images have totally different gray-level histograms. Accordingly, their nonextensive parameters are also very different from each other, as shown in Table 1.

**Figure 8:**
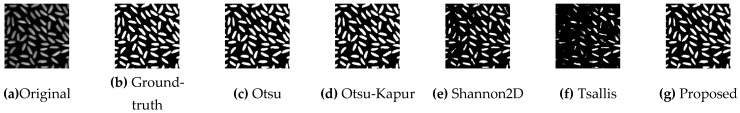
Rice.

**Figure 9:**
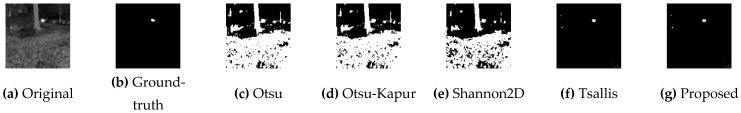
Infrared image.

**Figure 10:**
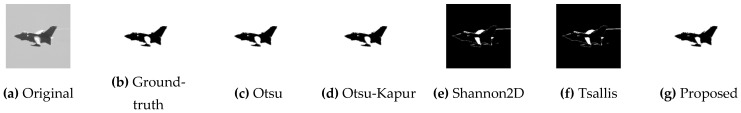
Jet.

**Figure 11:**
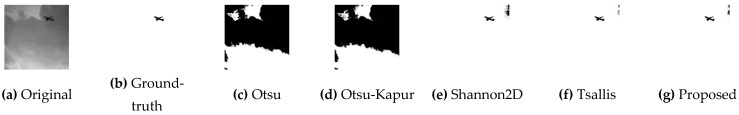
Plane.

**Figure 12:**
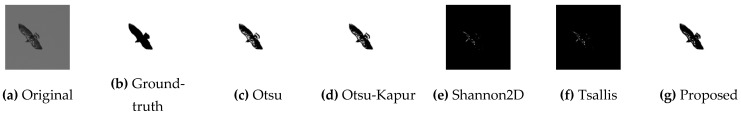
Hawk.

In order to further illustrate the segmentation results visually, we chose pictures 1–5 of Table 1 as examples, as shown in Figure 8, Figure 9, Figure 10, Figure 11 and Figure 12.

In Figure 8, the ground-truth image shows that the number of pixels in the foreground is comparable to that of background. Therefore, both the Otsu algorithm and the proposed algorithm achieve acceptable results. However, the entropy-based algorithms cannot yield good results, as shown in Figure 8e,f. In Figure 9, the infrared object is tiny in comparison with the full image size. The Otsu-based algorithm failed to determine the correct results as expected, as shown in Figure 9c,d. By comparison, the infrared image may have a long-range correlation among the pixels, so the Shannon entropy-based algorithm also failed, as shown in Figure 9e. The results of Figure 9f,g are very close to the ground-truth image, which indicates that the value of the nonextensive parameter *q* can correctly evaluate the long-range correlation in an image. In addition, the value of *q* is automatically generated by maximizing Equation (27), so the new algorithm is self-adaptive. The results of Figure 10 and Figure 12 are quite similar to that of Figure 8, since there is a large amount of noise in the background and the entropy-based algorithms are very unstable to perturbation, in spite of the increasing dimension of the histogram. However, the new algorithm can still correctly segment the images, which shows the potential application in a more general scope, including tiny object recognition (Figure 9 and Figure 11), background noise suppression (Figure 10 and Figure 12), and detection of long-range correlation.

From Figure 8e to Figure 12e, we can see that the 2-D Shannon algorithm, as a well-known entropic thresholding procedure, does not have stable outputs. However, the idea of extending the dimension of the histogram using the correlation of the neighboring pixels is still heuristic. It is of great interest to extend Equation (29) into two, or even higher, dimensions of the histogram because the development of optimization algorithms [15], refs. [19,20] can effectively reduce the computational cost.

For each image from the testing set, it should be mentioned that the new algorithm and the Tsallis entropy algorithm share the same value of *q*, which is determined by maximizing the information redundancy. However, the Tsallis entropy algorithm is very unstable if the image is subject to noise interference, even with a proper value of *q*. In comparison, the new algorithm is always stable. By comparison, Otsu algorithm is robust but cannot effectively recognize tiny objects. The new algorithm extracts the advantages of both the Otsu and entropy-based algorithms in a proper manner, and this point can be further shown using the detailed quality indices.

For 50 images in the testing set, Table 2, Table 3, Table 4 and Table 5 list the above-mentioned four quality indices of the results generated by the five different algorithms, respectively. Due to the variety in the testing set, the new algorithm cannot always ensure the best performance for all images, but its results are still acceptable. Furthermore, the statistical results of Table 2, Table 3, Table 4 and Table 5 clearly show the universality of the proposed algorithm for different kinds of images.

**Figure 13:**
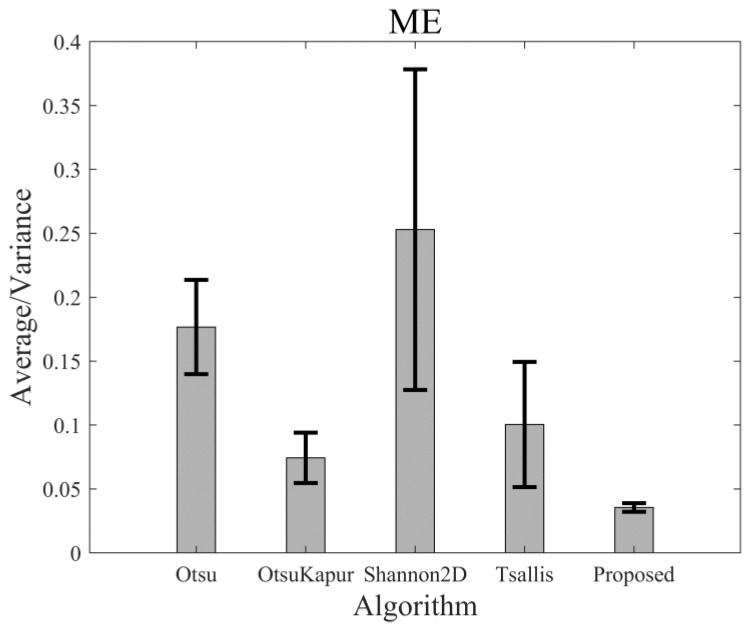
The average values of ME of different algorithms and the corresponding variance bars.

**Figure 14:**
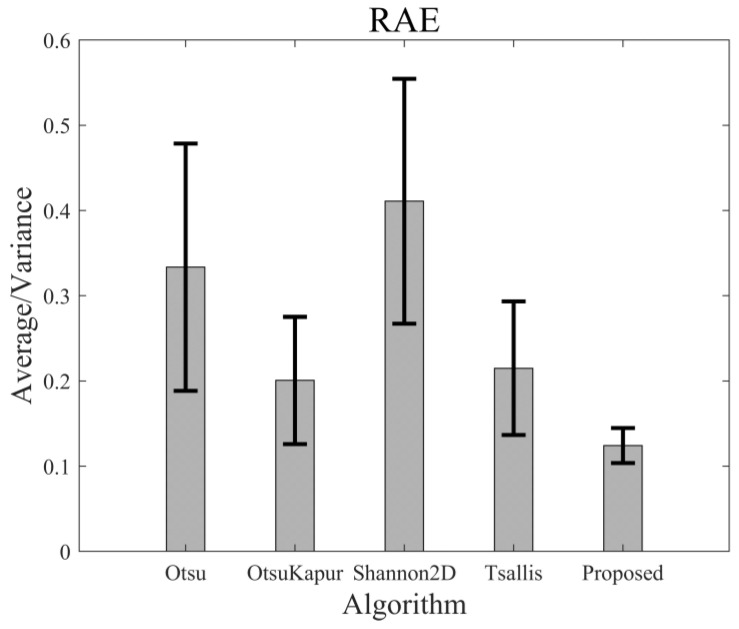
The average values of RAE of different algorithms and the corresponding variance bars.

For 50 testing images, the segmented results and the corresponding ME of the different thresholding algorithms may be very different. However, the performances of these algorithms can be statistically evaluated. Figure 13 shows the average ME values of the 50 images yielded by the above-mentioned five algorithms, and Figure 14 represents the average values of RAE. As shown, the average ME value of the proposed algorithm is the lowest in comparison with those of the other algorithms. Furthermore, the variance in the ME value of the proposed algorithm is much less than those of the other algorithms. Therefore, both accuracy (lower ME value) and stability (lower variance) of the new algorithm are better than those of the algorithms, which means that this algorithm is suitable and more robust for a more general category of images. By the same analysis, we can see that the average value and variance in RAE of the new algorithm were both the lowest among all the results, which indicates that the new algorithm is better than the others in foreground area detection.

**Figure 15:**
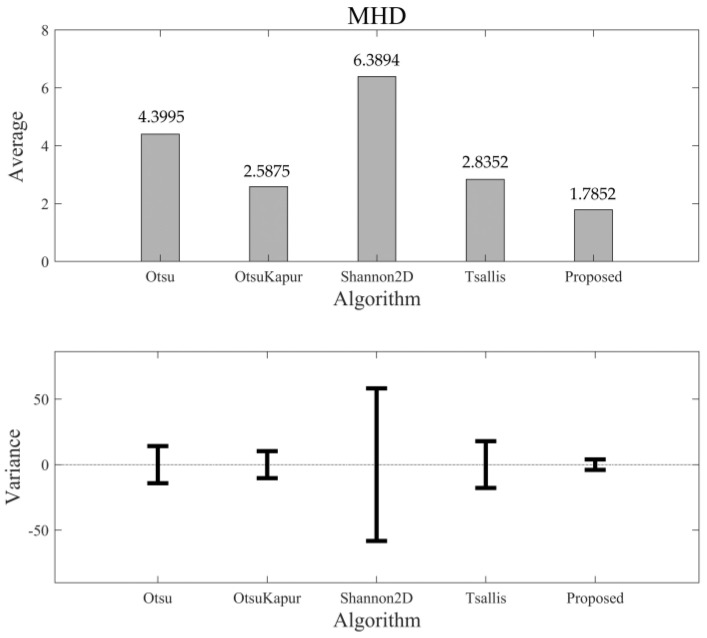
The average value of MHD of different algorithms and the corresponding variance bars.

**Figure 16:**
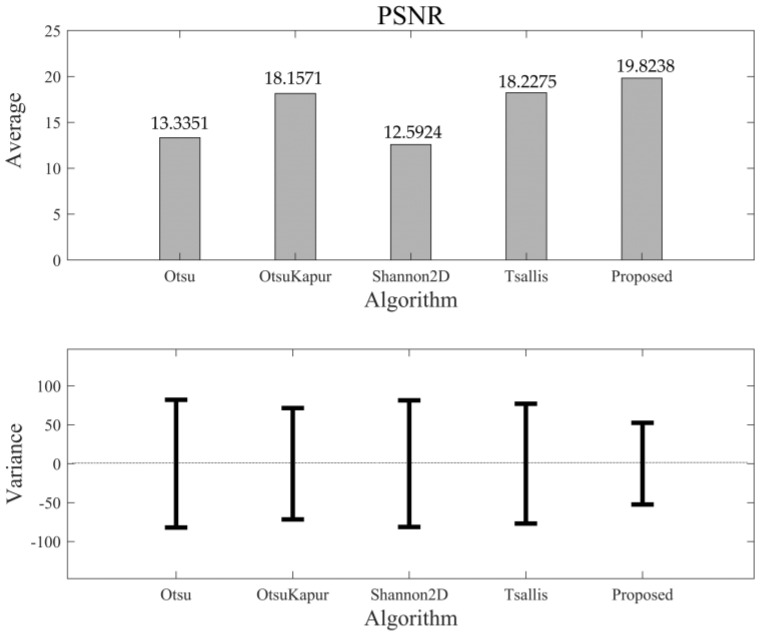
The average value of PSNR of different algorithms and the corresponding variance bars.

As mentioned above, the values of MHD and PSNR are not normalized. For 50 segmented results of the testing set, the distributions of MHD and PSNR are not at [0,1], but at (0,∞). Therefore, it is possible that their variances are larger than the averages. Figure 15 shows the comparison of MHD among the five algorithms. As we can see, the average MHD of the new algorithm again achieves the lowest value, and the corresponding variance is less than that of the others. This means that the new algorithm can maintain the shape of the objects in a more correct and stable manner. Figure 16 shows the comparison results of PSNR. Unlike the other three quality indices, the larger PSNR value means a better quality of information transmission. Therefore, we can see that the new algorithm still performs better than the others in this quality index (largest PSNR value), with a better robustness (lowest variance).

## 5. Conclusions

In the task of computer vision, it is of great importance to explore algorithms that can correctly recognize the objects from different kinds of backgrounds in a stable way. The Otsu algorithm is based on the variance in the gray-level distribution of an image. It can yield stable thresholding results but has deficiencies in small target recognition. The entropy-based algorithms are suitable for small target extraction and can even detect the long-range correlation among pixels using a nonextensive parameter. However, the entropy-based objective functions can be easily disturbed by noise. In the present paper, based on the rigorous mathematical and numerical results, we combine the advantages of the Otsu algorithm and nonextensive entropy algorithm to develop a new algorithm that can effectively segment the objects from various kinds of background in a more stable manner. For 50 images chosen from different categories, the quality indices of ME, RAE, MHD, and PSNR were adopted to evaluate the segmentation results. In comparison with the other famous thresholding algorithms, the statistical results show that the proposed algorithm has better performance than the others in each of the four quality indices. In addition, there is no artificial intervention during the whole process. Therefore, the proposed algorithm is an approach to automatic image thresholding that has potential application in self-adaptive object recognition.

## Figures and Tables

**Table 1:** The values of *q* of 50 test images.

Test	*q*	Test	*q*	Test	*q*
1	0.6971	18	0.4908	35	0.5385
2	0.3891	19	0.3971	36	0.5217
3	0.6992	20	0.6089	37	0.5613
4	0.4067	21	0.5240	38	0.4409
5	0.6424	22	0.4844	39	0.5170
6	0.4933	23	0.4003	40	0.5139
7	0.4472	24	0.5079	41	0.5189
8	0.4706	25	0.4895	42	0.4960
9	0.4846	26	0.4724	43	0.5670
10	0.4990	27	0.5000	44	0.5107
11	0.5218	28	0.5730	45	0.5304
12	0.5159	29	0.5602	46	0.4680
13	0.4993	30	0.4379	47	0.4757
14	0.4572	31	0.5881	48	0.5823
15	0.6161	32	0.5557	49	0.5716
16	0.5976	33	0.4936	50	0.4884
17	0.5276	34	0.5479		

**Table 2:** The values of ME of 50 testing images segmented by 5 different algorithms.

Images	Otsu	Otsu-Kapur	Shannon2D	Tsallis	Proposed
1	3.628 × 10^−3^	8.611 × 10^−3^	1.213 × 10^−1^	2.205 × 10^−1^	4.408 × 10^−2^
2	5.453 × 10^−1^	5.366 × 10^−1^	4.563 × 10^−1^	1.487 × 10^−3^	1.416 × 10^−3^
3	2.946 × 10^−3^	2.188 × 10^−3^	8.968 × 10^−1^	8.965 × 10^−1^	3.899 × 10^−3^
4	6.011 × 10^−1^	6.169 × 10^−1^	6.639 × 10^−3^	1.514 × 10^−3^	2.614 × 10^−3^
5	1.083 × 10^−2^	9.282 × 10^−3^	9.399 × 10^−1^	9.401 × 10^−1^	4.328 × 10^−3^
6	3.580 × 10^−1^	3.136 × 10^−1^	1.053 × 10^−2^	1.247 × 10^−2^	2.623 × 10^−2^
7	4.384 × 10^−1^	1.006 × 10^−3^	1.006 × 10^−3^	1.822 × 10^−3^	1.388 × 10^−3^
8	2.930 × 10^−1^	5.017 × 10^−3^	1.941 × 10^−2^	5.503 × 10^−3^	5.017 × 10^−3^
9	1.168 × 10^−3^	1.917 × 10^−3^	3.372 × 10^−3^	3.196 × 10^−3^	2.799 × 10^−3^
10	7.516 × 10^−3^	1.917 × 10^−3^	9.763 × 10^−3^	9.532 × 10^−3^	7.789 × 10^−3^
11	2.305 × 10^−2^	3.689 × 10^−2^	5.975 × 10^−2^	3.689 × 10^−2^	3.689 × 10^−2^
12	2.034 × 10^−2^	3.025 × 10^−2^	5.327 × 10^−2^	3.943 × 10^−2^	3.943 × 10^−2^
13	1.950 × 10^−2^	6.446 × 10^−3^	5.553 × 10^−2^	8.635 × 10^−1^	1.950 × 10^−2^
14	3.745 × 10^−1^	1.221 × 10^−2^	2.893 × 10^−2^	1.317 × 10^−2^	1.221 × 10^−2^
15	1.002 × 10^−2^	1.122 × 10^−2^	3.156 × 10^−2^	2.614 × 10^−2^	1.685 × 10^−2^
16	4.359 × 10^−3^	4.359 × 10^−3^	2.533 × 10^−2^	1.671 × 10^−2^	6.512 × 10^−3^
17	2.238 × 10^−2^	2.651 × 10^−2^	5.562 × 10^−2^	2.866 × 10^−2^	2.651 × 10^−2^
18	4.041 × 10^−1^	3.276 × 10^−2^	5.220 × 10^−2^	2.166 × 10^−2^	3.276 × 10^−2^
19	4.014 × 10^−1^	4.014 × 10^−1^	5.303 × 10^−4^	3.409 × 10^−4^	2.272 × 10^−4^
20	2.714 × 10^−1^	6.770 × 10^−4^	8.680 × 10^−4^	7.118 × 10^−2^	6.770 × 10^−4^
21	1.126 × 10^−2^	1.126 × 10^−2^	1.119 × 10^−2^	1.126 × 10^−2^	9.982 × 10^−3^
22	5.111 × 10^−1^	2.359 × 10^−2^	4.783 × 10^−2^	2.476 × 10^−2^	2.359 × 10^−2^
23	5.150 × 10^−1^	5.150 × 10^−1^	3.889 × 10^−1^	8.214 × 10^−3^	4.829 × 10^−3^
24	4.171 × 10^−1^	4.278 × 10^−2^	4.346 × 10^−2^	4.391 × 10^−2^	4.278 × 10^−2^
25	5.128 × 10^−1^	3.313 × 10^−3^	7.931 × 10^−3^	3.313 × 10^−3^	3.313 × 10^−3^
26	1.737 × 10^−3^	6.830 × 10^−4^	6.803 × 10^−3^	2.590 × 10^−3^	2.590 × 10^−3^
27	1.998 × 10^−2^	2.161 × 10^−2^	1.662 × 10^−2^	1.998 × 10^−2^	2.161 × 10^−2^
28	4.378 × 10^−2^	5.334 × 10^−2^	1.167 × 10^−1^	6.683 × 10^−2^	5.843 × 10^−2^
29	3.967 × 10^−1^	2.186 × 10^−2^	3.360 × 10^−2^	1.654 × 10^−2^	2.186 × 10^−2^
30	4.126 × 10^−1^	6.240 × 10^−4^	9.885 × 10^−1^	8.053 × 10^−4^	1.888 × 10^−3^
31	9.050 × 10^−3^	1.214 × 10^−2^	1.954 × 10^−2^	2.059 × 10^−2^	1.749 × 10^−2^
32	1.881 × 10^−1^	1.928 × 10^−1^	8.684 × 10^−1^	2.020 × 10^−1^	1.975 × 10^−1^
33	2.676 × 10^−1^	2.789 × 10^−1^	2.642 × 10^−1^	2.921 × 10^−1^	2.882 × 10^−1^
34	5.120 × 10^−3^	5.020 × 10^−3^	9.320 × 10^−1^	7.235 × 10^−3^	6.085 × 10^−3^
35	3.980 × 10^−2^	8.950 × 10^−3^	1.796 × 10^−2^	9.851 × 10^−3^	9.851 × 10^−3^
36	9.672 × 10^−2^	8.409 × 10^−2^	8.336 × 10^−2^	8.409 × 10^−2^	8.409 × 10^−2^
37	1.425 × 10^−2^	1.195 × 10^−2^	9.081 × 10^−1^	7.309 × 10^−3^	6.360 × 10^−3^
38	2.487 × 10^−1^	3.413 × 10^−4^	9.976 × 10^−1^	2.453 × 10^−4^	3.413 × 10^−4^
39	7.105 × 10^−3^	4.132 × 10^−3^	9.818 × 10^−1^	3.855 × 10^−3^	3.855 × 10^−3^
40	2.120 × 10^−1^	1.879 × 10^−1^	5.034 × 10^−1^	5.036 × 10^−1^	1.434 × 10^−1^
41	1.169 × 10^−1^	1.243 × 10^−1^	2.059 × 10^−1^	1.661 × 10^−1^	1.462 × 10^−1^
42	5.753 × 10^−3^	2.372 × 10^−3^	9.867 × 10^−1^	2.372 × 10^−3^	3.891 × 10^−3^
43	1.121 × 10^−1^	1.172 × 10^−1^	1.540 × 10^−1^	1.158 × 10^−1^	1.172 × 10^−1^
44	1.081 × 10^−4^	2.012 × 10^−3^	7.158 × 10^−1^	2.792 × 10^−3^	3.765 × 10^−3^
45	6.593 × 10^−2^	7.443 × 10^−2^	7.850 × 10^−2^	7.610 × 10^−2^	7.747 × 10^−2^
46	4.632 × 10^−1^	2.564 × 10^−3^	8.158 × 10^−3^	2.913 × 10^−3^	2.564 × 10^−3^
47	1.810 × 10^−1^	1.621 × 10^−1^	1.746 × 10^−1^	1.447 × 10^−1^	1.563 × 10^−1^
48	1.727 × 10^−2^	1.940 × 10^−2^	2.483 × 10^−1^	3.504 × 10^−2^	2.738 × 10^−2^
49	6.357 × 10^−3^	4.805 × 10^−3^	4.645 × 10^−3^	1.680 × 10^−3^	1.226 × 10^−3^
50	1.773 × 10^−1^	1.574 × 10^−3^	1.504 × 10^−3^	1.875 × 10^−3^	1.574 × 10^−3^

**Table 3:** The value of RAE of 50 testing images segmented by 5 different algorithms.

Images	Otsu	Otsu-Kapur	Shannon2D	Tsallis	Proposed
1	1.031 × 10^−2^	2.776 × 10^−2^	4.025 × 10^−1^	7.316 × 10^−1^	1.462 × 10^−1^
2	9.964 × 10^−1^	9.963 × 10^−1^	9.957 × 10^−1^	3.640 × 10^−1^	3.428 × 10^−1^
3	1.988 × 10^−3^	9.748 × 10^−4^	9.758 × 10^−1^	9.753 × 10^−1^	3.780 × 10^−3^
4	2.897 × 10^−1^	2.653 × 10^−1^	9.690 × 10^−1^	9.694 × 10^−1^	2.158 × 10^−1^
5	6.043 × 10^−1^	6.202 × 10^−1^	6.624 × 10^−3^	1.380 × 10^−3^	2.511 × 10^−3^
6	3.668 × 10^−1^	4.187 × 10^−1^	1.045 × 10^−2^	1.459 × 10^−2^	3.068 × 10^−2^
7	9.851 × 10^−1^	2.551 × 10^−2^	2.051 × 10^−2^	1.415 × 10^−1^	8.173 × 10^−2^
8	9.622 × 10^−1^	3.009 × 10^−1^	6.280 × 10^−1^	3.210 × 10^−1^	3.009 × 10^−1^
9	3.699 × 10^−2^	8.886 × 10^−2^	1.464 × 10^−1^	1.398 × 10^−1^	1.246 × 10^−1^
10	1.569 × 10^−2^	8.886 × 10^−2^	1.352 × 10^−2^	9.160 × 10^−3^	3.171 × 10^−3^
11	2.542 × 10^−2^	4.068 × 10^−2^	6.589 × 10^−2^	4.068 × 10^−2^	4.068 × 10^−2^
12	2.290 × 10^−2^	3.416 × 10^−2^	6.015 × 10^−2^	4.452 × 10^−2^	4.452 × 10^−2^
13	8.167 × 10^−2^	2.120 × 10^−1^	4.338 × 10^−1^	9.225 × 10^−1^	2.120 × 10^−1^
14	9.742 × 10^−1^	5.519 × 10^−1^	7.448 × 10^−1^	5.705 × 10^−1^	5.519 × 10^−1^
15	1.710 × 10^−2^	3.738 × 10^−2^	1.961 × 10^−1^	1.609 × 10^−1^	9.227 × 10^−2^
16	1.156 × 10^−2^	1.156 × 10^−2^	6.296 × 10^−2^	4.434 × 10^−2^	1.727 × 10^−2^
17	1.011 × 10^−1^	1.175 × 10^−1^	2.184 × 10^−1^	1.258 × 10^−1^	1.175 × 10^−1^
18	4.710 × 10^−1^	2.131 × 10^−2^	4.327 × 10^−2^	7.205 × 10^−3^	2.131 × 10^−2^
19	9.948 × 10^−1^	9.948 × 10^−1^	2.029 × 10^−1^	1.129 × 10^−1^	6.779 × 10^−2^
20	9.675 × 10^−1^	2.416 × 10^−2^	3.314 × 10^−2^	3.136 × 10^−2^	2.416 × 10^−2^
21	4.315 × 10^−1^	4.315 × 10^−1^	4.300 × 10^−1^	4.315 × 10^−1^	4.021 × 10^−1^
22	9.552 × 10^−1^	4.787 × 10^−1^	6.642 × 10^−1^	4.938 × 10^−1^	4.787 × 10^−1^
23	9.762 × 10^−1^	9.762 × 10^−1^	9.687 × 10^−1^	3.959 × 10^−1^	2.781 × 10^−1^
24	8.857 × 10^−1^	4.430 × 10^−1^	4.469 × 10^−1^	4.494 × 10^−1^	4.430 × 10^−1^
25	9.551 × 10^−1^	1.143 × 10^−1^	2.476 × 10^−1^	1.208 × 10^−1^	1.143 × 10^−1^
26	1.790 × 10^−3^	1.692 × 10^−5^	6.459 × 10^−3^	2.008 × 10^−3^	2.008 × 10^−3^
27	1.010 × 10^−2^	1.261 × 10^−2^	6.825 × 10^−3^	1.010 × 10^−2^	1.261 × 10^−2^
28	3.615 × 10^−2^	1.110 × 10^−1^	3.536 × 10^−1^	1.782 × 10^−1^	1.395 × 10^−1^
29	4.042 × 10^−1^	2.227 × 10^−2^	3.423 × 10^−2^	1.685 × 10^−2^	2.227 × 10^−2^
30	4.145 × 10^−1^	4.988 × 10^−4^	9.944 × 10^−1^	2.199 × 10^−4^	9.230 × 10^−4^
31	1.719 × 10^−2^	1.781 × 10^−2^	9.285 × 10^−2^	8.773 × 10^−2^	6.442 × 10^−2^
32	1.937 × 10^−1^	1.988 × 10^−1^	9.007 × 10^−1^	2.088 × 10^−1^	2.038 × 10^−1^
33	2.787 × 10^−1^	2.904 × 10^−1^	2.752 × 10^−1^	3.042 × 10^−1^	3.001 × 10^−1^
34	3.808 × 10^−3^	2.893 × 10^−3^	9.977 × 10^−1^	3.324 × 10^−3^	1.986 × 10^−3^
35	1.867 × 10^−1^	4.910 × 10^−2^	6.479 × 10^−2^	2.067 × 10^−2^	2.067 × 10^−2^
36	2.157 × 10^−1^	1.399 × 10^−1^	9.805 × 10^−2^	1.399 × 10^−1^	1.399 × 10^−1^
37	1.514 × 10^−2^	1.264 × 10^−2^	9.946 × 10^−1^	2.725 × 10^−3^	5.820 × 10^−3^
38	2.491 × 10^−1^	3.419 × 10^−4^	9.995 × 10^−1^	2.457 × 10^−4^	3.419 × 10^−4^
39	7.191 × 10^−3^	4.195 × 10^−3^	9.986 × 10^−1^	2.747 × 10^−3^	2.747 × 10^−3^
40	1.141 × 10^−2^	9.791 × 10^−3^	9.979 × 10^−1^	9.987 × 10^−1^	4.589 × 10^−3^
41	1.679 × 10^−1^	1.796 × 10^−1^	3.030 × 10^−1^	2.432 × 10^−1^	2.133 × 10^−1^
42	5.796 × 10^−3^	1.452 × 10^−3^	9.996 × 10^−1^	1.452 × 10^−3^	4.138 × 10^−4^
43	1.128 × 10^−1^	1.190 × 10^−1^	1.599 × 10^−1^	1.174 × 10^−1^	1.190 × 10^−1^
44	1.108 × 10^−4^	2.063 × 10^−3^	7.342 × 10^−1^	2.864 × 10^−3^	3.862 × 10^−3^
45	5.195 × 10^−2^	6.429 × 10^−2^	7.306 × 10^−2^	6.650 × 10^−2^	6.842 × 10^−2^
46	9.915 × 10^−1^	3.928 × 10^−1^	6.730 × 10^−1^	4.237 × 10^−1^	3.928 × 10^−1^
47	2.117 × 10^−1^	1.897 × 10^−1^	2.042 × 10^−1^	1.693 × 10^−1^	1.829 × 10^−1^
48	4.399 × 10^−2^	2.698 × 10^−2^	5.420 × 10^−1^	1.175 × 10^−1^	8.164 × 10^−2^
49	6.647 × 10^−3^	4.985 × 10^−3^	4.885 × 10^−3^	1.077 × 10^−3^	3.077 × 10^−4^
50	8.989 × 10^−1^	6.968 × 10^−2^	4.994 × 10^−2^	1.045 × 10^−2^	6.968 × 10^−2^

**Table 4:** The value of MHD of 50 testing images segmented by 5 different algorithms.

Images	Otsu	Otsu-Kapur	Shannon2D	Tsallis	Proposed
1	0.7029	1.2836	4.9663	6.7715	3.1038
2	11.7156	11.6110	10.6244	0.1976	0.1890
3	0.5922	0.5024	18.4577	18.4577	0.6871
4	14.3347	14.6405	0.8646	0.3246	0.4621
5	1.2517	1.1626	21.3836	21.3640	0.7552
6	9.2300	10.2477	1.0750	0.9396	1.4021
7	9.4387	0.1241	0.1157	0.2332	0.1869
8	7.9342	0.5524	1.4978	0.5838	0.5524
9	0.1166	0.2286	0.3417	0.3543	0.3202
10	1.0453	1.0482	1.2970	1.3117	1.0921
11	1.3459	1.8848	2.5976	1.8848	1.8848
12	1.1320	1.5287	2.3010	1.9039	1.9039
13	0.3694	0.8507	1.6909	8.8287	0.8507
14	8.6337	1.3737	2.2707	1.4357	1.3737
15	0.8680	0.9532	1.6164	1.5651	1.2187
16	0.2710	0.2710	1.0297	0.7990	0.3800
17	1.2400	1.3689	2.0293	1.4668	1.3689
18	7.9769	1.9261	2.4025	1.5822	1.9261
19	6.9689	6.9689	0.0219	0.0339	0.0294
20	4.1651	0.1266	0.1433	0.1349	0.1266
21	0.8545	0.8545	0.8520	0.8545	0.7773
22	9.8265	1.6602	2.3906	1.6995	1.6602
23	11.2817	11.2817	9.7391	0.8620	0.5391
24	6.1740	1.8942	1.9034	1.9199	1.8942
25	9.1926	0.4914	1.0172	0.4914	0.4914
26	0.3106	0.1967	1.0152	0.5496	0.5496
27	1.8218	1.8572	1.6733	1.8218	1.8572
28	3.2017	3.4556	1.8630	3.4965	3.4838
29	4.6476	0.4281	0.5962	0.3106	0.4281
30	10.3385	0.1780	21.6996	0.2272	0.3698
31	1.2804	1.6457	2.2450	2.4751	2.1817
32	5.6362	5.7402	18.5767	5.9757	5.8568
33	5.5016	6.1290	5.3075	6.9198	6.7114
34	0.7113	0.7200	21.2428	1.2786	1.0295
35	3.6282	1.2932	2.0579	1.6302	1.6302
36	5.9186	5.4392	5.6658	5.4392	5.4392
37	1.8822	1.6910	20.8121	1.2489	1.0532
38	10.7641	0.0966	22.2088	0.0760	0.0966
39	0.6336	0.4747	21.8053	0.5334	0.5334
40	0.5525	0.2500	20.0422	0.7332	0.7332
41	4.2936	4.5829	6.5464	5.7988	5.2712
42	0.4311	0.3480	22.1219	0.3480	0.5231
43	7.3737	7.5572	8.7283	7.5083	7.5572
44	0.0488	0.4478	17.6588	0.5756	0.7164
45	4.3665	4.8212	4.9057	4.8945	4.9490
46	8.5651	0.2903	0.7585	0.3222	0.2903
47	8.3824	7.9125	7.8823	7.4598	7.7639
48	1.4807	1.8018	7.0569	2.4557	2.1798
49	0.6611	0.5477	0.8580	0.4113	0.3190
50	5.5122	0.1284	0.1600	0.2234	0.1284

**Table 5:** The value of PSNR of 50 testing images segmented by 5 different algorithms.

Images	Otsu	Otsu-Kapur	Shannon2D	Tsallis	Proposed
1	24.4028	20.6494	9.1613	6.5658	13.5576
2	2.6335	2.7034	3.4067	28.2768	28.4887
3	25.3066	26.5994	0.4728	0.4745	25.0903
4	2.2099	2.0973	21.7790	28.1972	25.8268
5	19.6514	20.3235	0.2688	0.2679	23.6369
6	19.4818	19.4818	19.5086	19.4818	20.0075
7	3.5809	29.9699	29.9699	27.3923	28.5733
8	5.3313	22.9952	17.1198	22.5936	22.9952
9	29.3248	27.1724	24.7207	24.9539	25.5296
10	21.2398	21.2398	20.1041	20.2081	21.0849
11	16.3721	14.3309	12.2365	14.3309	14.3309
12	16.9161	15.1918	12.7347	14.0417	14.0417
13	21.9069	17.0987	12.5542	0.6371	17.0987
14	4.2650	19.1309	15.3857	18.8041	19.1309
15	19.9912	19.4977	15.0084	15.8254	17.7334
16	23.6062	23.6062	15.9631	17.7682	21.8623
17	16.5001	15.7653	12.5471	15.4270	15.7653
18	3.9349	14.8456	12.8232	16.6430	14.8456
19	3.9638	3.9638	32.7548	34.6736	36.4345
20	5.6638	31.6936	30.6145	31.4764	31.6936
21	21.9069	17.0987	12.5542	0.6371	17.0987
22	2.9149	16.2726	13.2023	16.0618	16.2726
23	19.9912	19.4977	15.0084	15.8254	17.7334
24	23.6062	23.6062	15.9631	17.7682	21.8623
25	16.5001	15.7653	12.5471	15.4270	15.7653
26	27.6002	31.6554	21.6728	25.8667	25.8667
27	16.9925	16.6516	17.7922	16.9925	16.6516
28	13.5864	12.7288	9.3287	11.7501	12.3330
29	4.0145	16.6031	14.7356	17.8129	16.6031
30	3.8442	32.0482	0.0499	30.9402	27.2400
31	20.4332	19.1545	17.0905	16.8619	17.5713
32	7.2547	7.1474	0.6125	6.9447	7.0439
33	5.7238	5.5455	5.7792	5.3433	5.4028
34	22.9073	22.9930	0.3057	21.4056	22.1574
35	14.0006	20.4814	17.4554	20.0652	20.0652
36	10.1444	10.7521	10.7900	10.7521	10.7521
37	18.4612	19.2256	0.4183	21.3612	21.9652
38	6.0426	34.6682	0.0101	36.1024	34.6682
39	21.4843	23.8383	0.0797	24.1388	24.1388
40	6.7362	7.2601	2.9806	2.9789	8.4326
41	9.3201	9.0553	6.8620	7.7954	8.3483
42	22.4005	26.2482	0.0582	26.2482	24.0984
43	9.5023	9.3082	8.1233	9.3610	9.3082
44	39.6614	26.9637	1.4516	25.5396	24.2415
45	11.8090	11.2821	11.0510	11.1858	11.1083
46	3.3420	25.9106	20.8839	25.3555	25.9106
47	7.4230	7.9009	7.5796	8.3931	8.0586
48	17.6259	17.1214	6.0497	14.5537	15.6255
49	21.9673	23.1828	23.3298	27.7469	29.1127
50	7.5114	28.0297	28.2257	27.2700	28.0297

## Data Availability

Data are contained within the article.
